# Fully Automatic Rapid inversion time (TI) Adjustment for Late Gadolinium Enhancement (LGE) Imaging Using a Pencil Beam Excitation Pulse for Single-Line T1 (SLT1) Mapping of Myocardium

**DOI:** 10.1186/1532-429X-18-S1-P322

**Published:** 2016-01-27

**Authors:** Wolfgang G Rehwald, David C Wendell, Elizabeth Jenista, Han W Kim, Michele Parker, Yutaka Natsuaki, Enn-Ling Chen, Igor Klem, Raymond Kim

**Affiliations:** 1 Siemens Healthcare, Chapel Hill, NC USA; 2 grid.189509.c0000000100241216Duke Cardiovascular MR Center, Duke University Medical Center, Durham, NC USA

## Background

LGE requires appropriate TI setting to null viable myocardium, which can be challenging and time consuming. Phase sensitive inversion recovery (PSIR) makes the TI choice less crucial for 2D imaging. However, for 3D PSIR navigator gating is required for both IR and reference data significantly increasing scan time. Additionally using a fixed single TI for a 3D scan while contrast washes out is subpotimal and worsens contrast-to-noise ratio (CNR). If repeated T1 assessment was possible during 3D LGE, scan time could be reduced and CNR optimized. Although TI scout and T1 mapping sequences could be used to determine correct TI, they cannot be executed rapidly and repeatedly during 3D LGE. We developed a rapid SLT1 technique that can be executed repeatedly during 3D or before 2D scans with minimal time loss. It calculates and writes the TI setting into the protocol automatically.

## Methods

**Figure 1:**
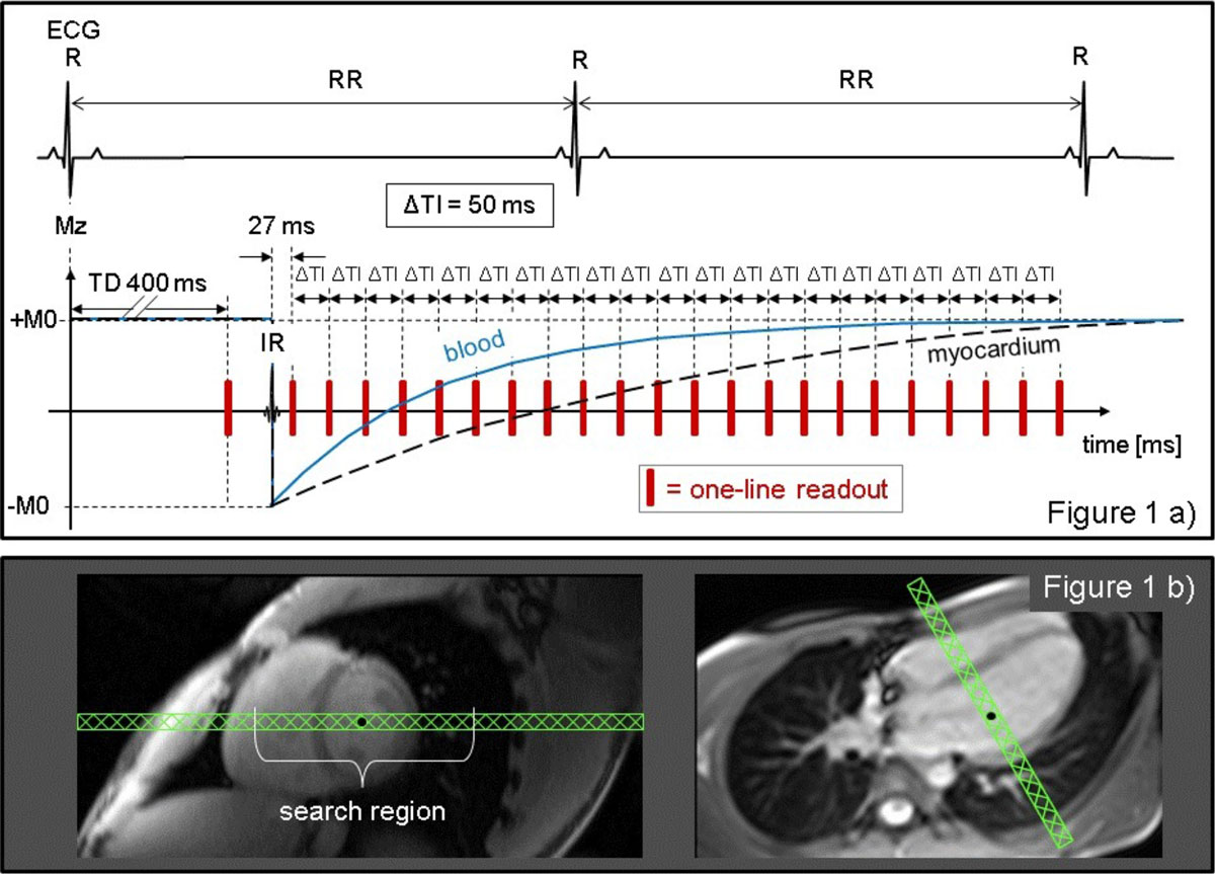
Figure 1

A pulse sequence including a single spatially non-selective IR pulse and multiple pencil beam excitation pulses (20 × 20 mm, α = 12°) for creating gradient echoes was implemented and tested in 17 post-contrast (POST) and 10 pre-contrast (PRE) free breathing patients on a 1.5T MAGNETOM Avanto (Siemens). The first echo was acquired before the IR pulse, 400 ms after the R-wave (Figure 1a). After the IR, data were read out every 50 ms for 2s (POST) or 3s (PRE). The pencil beam was placed along the intersection of a mid-ventricular short-axis and the 4 chamber view, with its center in the LV cavity (Figure 1b). T1 values were calculated per pixel by a least squares trust region algorithm. Within the search region, starting at the LV center and advancing right, an edge detection algorithm found the edges between LV, septum, and RV. Myocardial T1 was calculated as average T1 in the septal compartment; blood T1 as average T1 within LV center and the septal edge. A T1 map (MAP) was acquired as gold-standard. Comparisons were made by paired t-test.

**Figure 2:**
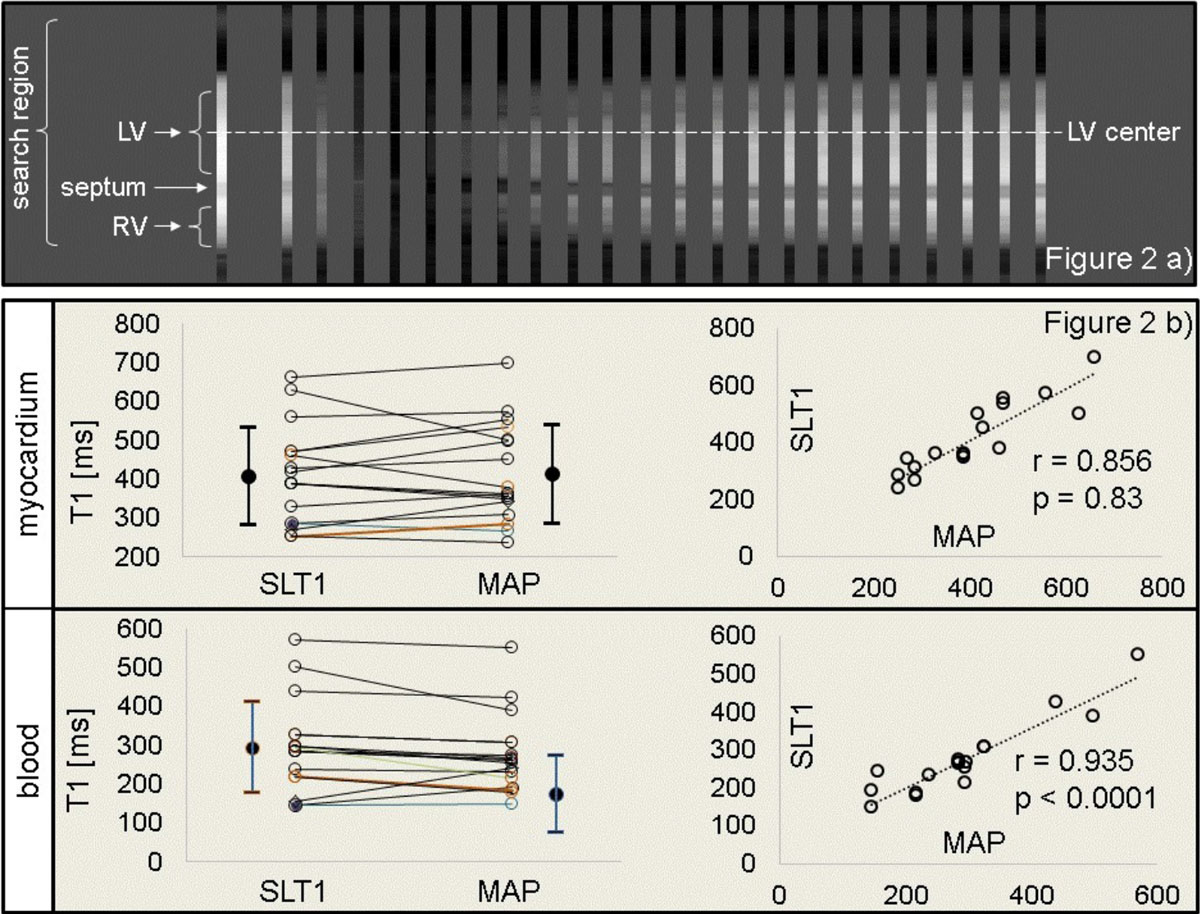
Figure 2

## Results

Figure 2a shows a typical set of single lines acquired POST plotted versus time, showing septum and blood pools. Their different T1 recovery and septal motion can be seen. By SLT1 and MAP respectively, POST myocardial T1 (mean ± SD, ms) was 415 ± 127 and 409 ± 125 and POST blood T1 was 176 ± 100 and 296 ± 117. T1 was identical (p > 0.05) in both myocardium and blood. PRE myocardial T1 was identical by SLT1 and MAP respectively (1031 ± 68 and 1039 ± 29, p > 0.05), whereas blood T1 was not (1488 ± 172 and 1641 ± 107, p < 0.05). Figure 2b shows POST T1 values of myocardium (top) and blood (bottom) for all patients. The matching value pairs (left graphs) illustrate the similarity of SLT1 and MAP values. The graphs on the right show the close correlation of SLT1 and MAP data.

## Conclusions

This simple, rapid, and fully automated adjustment technique can be used to calculate myocardial T1 and set the optimal myocardial TI for LGE scans. It allows quick TI adjustment for 2D and readjustment during 3D scans. The technique is repeatable and the beam can be placed automatically without operator intervention.

